# Cognition in MS correlates with resting-state oscillatory brain activity: An explorative MEG source-space study^☆^

**DOI:** 10.1016/j.nicl.2013.05.003

**Published:** 2013-05-13

**Authors:** M.L. Van der Meer, P. Tewarie, M.M. Schoonheim, L. Douw, F. Barkhof, C.H. Polman, C.J. Stam, A. Hillebrand

**Affiliations:** a Department of Neurology, Neuroscience Campus Amsterdam, VU University Medical Center, Amsterdam, The Netherlands; b Department of Clinical Neurophysiology and Magnetoencephalography Center, Neuroscience Campus Amsterdam, VU University Medical Center, Amsterdam, The Netherlands; c Department of Radiology and Nuclear Medicine, Neuroscience Campus Amsterdam, VU University Medical Center, Amsterdam, The Netherlands; d Departments of Anatomy and Neuroscience (Division of Clinical Neuroscience), Neuroscience Campus Amsterdam, VU University Medical Center, Amsterdam, The Netherlands

**Keywords:** Multiple Sclerosis, Cognition, Resting-state MEG, Beamforming, Oscillatory activity, Atlas, Expanded Disability Status Scale

## Abstract

Clinical and cognitive dysfunction in Multiple Sclerosis (MS) is insufficiently explained by structural damage as identified by traditional magnetic resonance imaging (MRI) of the brain, indicating the need for reliable functional measures in MS. We investigated whether altered resting-state oscillatory power could be related to clinical and cognitive dysfunction in MS. MEG recordings were acquired using a 151-channel whole-head MEG system from 21 relapsing remitting MS patients and 17 healthy age-, gender-, and education-matched controls, using an eyes-closed no-task condition. Relative spectral power was estimated for 78 regions of interest, using an atlas-based beamforming approach, for classical frequency bands; delta, theta, alpha1, alpha2, beta and gamma. These cortical power estimates were compared between groups by means of permutation analysis and correlated with clinical disability (Expanded Disability Status Scale: EDSS), cognitive performance and MRI measures of atrophy and lesion load. Patients showed increased power in the alpha1 band and decreased power in the alpha2 band, compared to controls, mainly in occipital, parietal and temporal areas, confirmed by a lower alpha peak-frequency. Increased power in the alpha1 band was associated with worse overall cognition and especially with information processing speed. Our quantitative relative power analysis of MEG recordings showed abnormalities in oscillatory brain dynamics in MS patients in the alpha band. By applying source-space analyses, this study provides a detailed topographical view of abnormal brain activity in MS patients, especially localized to occipital areas. Interestingly, poor cognitive performance was related to high resting-state alpha1 power indicating that changes in oscillatory activity might be of value as an objective measure of disease burden in MS patients.

## Highlights

• MEG was recorded in relapsing remitting MS patients and healthy controls.
• Atlas-based MEG beamformer was used for anatomical mapping of neuronal activity.
• Increased power in alpha1 band in patients, associated with cognitive dysfunction.
• Decreased power in alpha2 band in patients, confirmed by lower alpha peak-frequency.

## 1 Introduction

Multiple Sclerosis (MS) is an acquired progressive neurological disease with a highly variable course, leading to both physical symptoms and cognitive impairment. Clinical and cognitive decline in MS is insufficiently explained by classical MRI measures such as lesion load or atrophy of the white matter (Barkhof, 2002). However axonal damage and demyelination in the gray matter seem to correlate with clinical and cognitive deficits in MS (Geurts and Barkhof, 2008).

In physiological conditions modulations in neuron population firing probability occur preferentially during a certain phase of the oscillatory activity (Schnitzler and Gross, 2005). Demyelination and axonal damage could lead to altered firing probability and therefore to altered oscillatory cortical activity in MS. Neurophysiological techniques, such as EEG and MEG, can be used to detect such changes in activity, as has been demonstrated for neurological diseases such as Alzheimer's disease (de Haan et al., 2008; Jeong, 2004; Stam et al., 2006), Parkinson's disease (Bosboom et al., 2006; Ponsen et al., 2013; Stoffers et al., 2007), low-grade glioma (Bosma et al., 2008), traumatic brain injury (Kumar et al., 2009), and stroke (van Putten and Tavy, 2004). Up to 70% of patients with MS suffer from cognitive impairment (Rao et al., 1991); attention, information processing speed and memory being the most commonly affected domains (Chiaravalloti and DeLuca, 2008). There is increasing evidence that changes in oscillatory brain activity may be related to cognitive dysfunction in neurological disease (Schnitzler and Gross, 2005; Stam and van Straaten, 2012; Uhlhaas and Singer, 2006). We therefore hypothesize that cognitive impairment in MS patients might be partially explained by pathological changes in oscillatory brain activity.

To date, literature on EEG or MEG in MS is scarce. Visual inspection of EEG recordings from MS patients revealed more focal EEG abnormalities (slow activity) in patients with relapses compared to patients with a progressive course (Feng, 1981). A 5-year follow-up study did not show a significant correlation between visual EEG abnormalities and neurological disability (Quattrini et al., 1981). Yet, another group used computerized spectral analysis to demonstrate a positive relation between patients' disability and increased theta power over the temporal regions and increased beta power over the frontal regions, where visual interpretation of the EEG failed to demonstrate any correlations (Colon et al., 1981). Power spectral density analysis of EEG data, obtained during an auditory oddball task, revealed increased power in beta and gamma bands (especially over midfrontal areas) in MS patients compared to healthy controls (Vazquez-Marrufo et al., 2008). Similarly, for a visuo-spatial task, more beta and gamma power was found over occipital and right-frontal regions in relapsing remitting MS patients compared to a group of healthy controls, but no differences were found in the high frequency bands during resting-state, nor were there any significant correlations between quantitative EEG (QEEG) scores and Expanded Disability Status Scale (EDSS) (Vazquez-Marrufo et al., 2008).

Compared to EEG, MEG provides a reference free method and the magnetic fields are much less disturbed by the skull. MS research using EEG and MEG has recently focussed on altered functional connectivity, which referrers to statistical interdependencies between physiological time series (Cover et al., 2006; Leocani et al., 2000; Schoonheim et al., 2013; Tecchio et al., 2008), and changes in functional network topology (Hardmeier et al., 2012; Schoonheim et al., 2013). A more basic characterization, including for example global and local spectral analysis of the rhythmic MEG activity in MS patients has not been performed to date. Yet, knowledge of changes in local spectral power seems fundamental in comprehending the outcome of connectivity research. Additionally, the aforementioned studies were performed at the sensor level, i.e. results were estimated based on the extracranial recordings directly, making interpretation of these results in terms of the specific anatomical brain regions that are involved more difficult. In addition, investigation of abnormal MEG activity at the source-level facilitates comparison with other neuroimaging techniques, notably structural and functional MRI.

The aim of the present MEG study was therefore to explore differences in resting-state oscillatory brain activity in MS patients compared to healthy controls, and to relate these differences to cognitive performance, physical disability and structural deficits measured with MRI. A recently developed technique, projecting sensor-based data onto an atlas-based source-space using beamforming, was applied (Hillebrand et al., 2012) in order to provide a detailed anatomical mapping of cortical rhythms for 78 regions of interest (ROIs).

## 2 Methods

### 2.1 General study design

In this cross-sectional study MS patients and healthy controls underwent MEG, MRI, neurological examination and neuropsychological assessment on the same day. Outcome measures were global relative power, relative power per ROI (regional relative power), peak frequency, anterior–posterior gradients, diffuse slow-wave activity and the presence of asymmetry. These outcome measures were associated with cognition and MRI measures.

### 2.2 Subject characteristics

MS patients and healthy volunteers were recruited from an ongoing large clinical study at the Multiple Sclerosis Center of the VU University Medical Center, as described in a previous MEG study in the same subjects (Schoonheim et al., 2013). Our project involved 34 MS patients (17 women, mean age 41.4 ± 8.0 years, disease duration 8.1 ± 1.6 years) and 28 healthy controls (14 women, mean age 39.8 ± 10.5 years), matched for age, gender and educational level (using a Dutch classification system ranging from 1 (only primary education) to 7 (university degree)). Twenty four participants were excluded from further analysis due to unavailability of an anatomical MRI (n = 2), failed MEG/MRI co-registration (n = 10) and too many artifacts in the raw MEG data (n = 12). Consequently, 21 MS patients (mean age 41.9 ± 7.7, disease duration 6.8 ± 0.9 years) and 17 controls (mean age 39.8 ± 9.8) remained in the present study, who were still gender-, age- and education-matched. Patients were diagnosed with MS according to the revised McDonald Criteria (Polman et al., 2005). None of the healthy controls suffered from a neurological or psychiatric disease, nor did they use any medication or drugs. Eight patients were treated with interferon β since diagnosis, one of them switched to glatiramer acetate and two to natalizumab, which they received during this study. No other medication was used. Patients were assessed according to a clinical protocol, involving history taking, neurological examination, blood tests, neuropsychological tests, MRI of the brain and MEG. Physical disability was measured using the Expanded Disability Status Scale (EDSS) (Kurtzke, 1983). The study protocol was approved by the Local Research Ethics Committee, whose ethics review criteria conformed to the Helsinki declaration. All subjects had given written informed consent prior to participation.

### 2.3 MRI

An MRI scan was obtained from all subjects, using a 3 T-MRI system (GE Signa HDXT V15m). 2D dual-echo T2-weighted sequence (TR 9680 ms, TE 22/112 ms) and T1-weighted sequence (TR 475 ms, TE 9 ms) were obtained with 48 slices of 3 mm and 3D-T1 heavily T1-weighted sequence (FSPGR, TR 7.8 ms, TE 3.0 ms, TI 450 ms) with 1 mm slices covering the entire brain. All scans were inspected by an experienced rater (MMS). T1-hypointense and T2-hyperintense lesions in MS patients were marked and their volumes were measured using a local-threshold technique. Total normal gray matter volume (NGMV), total normal white matter volume (NWMV), and normal whole brain volumes (NBV), corrected for head size, were estimated using FSPGR images and SIENAX (Smith et al., 2002) version 2.5 (part of FSL 4.1, FMRIB's Software Library, http://www.fmrib.ox.ac.uk/fsl). Thalamic volumes were outlined and volumes measured using FIRST (part of FSL), as described before for this cohort (Schoonheim et al., 2012). Left and right volumes were summed to give the total volume.

### 2.4 Neuropsychological evaluation

Cognitive function in all subjects was assessed according to the protocol used and described before (Schoonheim et al., 2012). A Brief Repeatable Battery of Neuropsychological Tests (BRB-N), the selective reminder test (SRT), the 10/36 spatial recall test (SPART), the symbol digit modalities test (SDMT), the word list generation test (WLG), the concept shifting test (CST), the Stroop color-word test and the memory comparison test (MCT) were administered. Individual patients' test scores were converted to z-scores, using the means and standard deviations of the entire group of participants. The z-scores for all tests were averaged for each subject, creating overall cognition z-score. Subsequently, individual scores on the tests were summarized into seven cognitive domains, namely (1) executive functioning (CST, WLG), (2) information processing speed (SDMT), (3) psychomotor speed (CST, SDMT), (4) attention (Stroop), (5) verbal memory (SRT), (6) working memory (MCT), and (7) visuospatial memory (SPART). Construction of these domains with comparable cognitive tests has been reported previously and was based on a principal component analysis using varimax rotation with Kaiser normalization performed on the z-scores for a large group of healthy controls (Klein et al., 2003), and these domains are commonly used in neurocognitive practice and research.

### 2.5 MEG recording

MEG data were acquired using a 151-channel whole-head MEG system (CTF Systems Inc., Port Coquitlam, BC, Canada), while subjects were seated inside a magnetically shielded room (Vacuum-schmelze GmbH, Hanau, Germany). A third-order software gradient (Vrba et al., 1999) was used with a recording passband of 0–150 Hz and a sample frequency of 625 Hz. At the beginning and end of the measurement, the head position relative to the coordinate system of the helmet was determined by leading small currents through three position coils situated at the left and right pre-auricular points and the nasion. Changes in head position smaller than 0.5 cm during the recording were accepted. The MEG recordings were performed in a no task, eyes-closed and eyes-open condition. Only data from the eyes-closed condition were analyzed here. For each participant, 5 min of the continuous resting-state, eyes-closed recording was divided into 45 epochs of 6.555 s. Channels and epochs were visually inspected. Epochs and channels were rejected based on system related artifacts (SQUID jumps, noisy, broken or saturated channels), physiological artifacts (eye movements, eye blinks, muscle artifacts), external artifacts (magnetized dental fillings) and environmental noise (Gross et al., 2013), as well as for representing an alert eyes-closed state, leading to discarding on average 5.7 channels (range: 2–14) and 8.4 epochs (range: 3–20). The selected epochs were subsequently projected to source-space.

### 2.6 Beamforming: time-series estimation for regions-of-interest

The technique used in this study was recently described (Hillebrand et al., 2012). A brief overview is given below. First, a subject's MRI was co-registered with the MEG data through identification of the same anatomical landmarks in the MRI that were also used for the placement of the MEG head-localization coils (i.e. left and right pre-auriculars and nasion). Only data from subjects where the estimated co-registration error was smaller than 0.8 cm were accepted for further analysis. The co-registered MRI was then spatially normalized to a template MRI using the SEG-toolbox in SPM8 (Friston et al., 2004). The new segmentation toolbox is an extension of the unified segmentation algorithm, which incorporates additional tissue priors for improved matching of the subject's MRI to the template (Ashburner and Friston, 2005; Weiskopf et al., 2011). The automated anatomical labeling (AAL) atlas was used to label the voxels in a subject's normalized co-registered MRI (Tzourio-Mazoyer et al., 2002). Subcortical structures were removed, and the voxels in the remaining 78 cortical ROIs were used for further analysis (Gong et al., 2009), after inverse transformation to the patient's co-registered MRI.

Neuronal activity for the labeled voxels in the ROIs was reconstructed using a beamforming approach known as Synthetic Aperture Magnetometry (SAM) (Robinson and Vrba, 1999). SAM works in a sequential fashion, where the activity for each voxel is reconstructed by selectively weighting the contribution from each MEG sensor to a voxel's time-series. This weighting is done such that the activity at a voxel is reconstructed without distortion, and at the same time the contribution from external (noise) sources is minimized (Hillebrand and Barnes, 2005; Hillebrand et al., 2005). The beamformer weights are based on the covariance of the data and the forward solution (lead field) of a dipolar source at the voxel location, where data were band-pass filtered from 0.5 to 48 Hz. To correct for non-uniform projection of sensor noise each beamformer weight was normalized by its vector norm. A time-window of, on average, 238 s (range: 164–282 s) was used for the computation of the data covariance matrix, which was considered sufficient for accurate estimation of the data covariance, and therefore for the accuracy of the reconstructed source power (Brookes et al., 2008). We used broadband data for the estimation of the beamformer weights, as this avoids overestimation of covariance between channels (Hillebrand and Barnes, 2005). The sensor-level data were subsequently projected through the beamformer weights, resulting in a time-series for each voxel. Each ROI contains many voxels and the number of voxels per ROI differed. In order to represent a ROI by a single time-series, we selected, for each ROI and frequency band separately, the voxel with maximum absolute power in that frequency band (Hillebrand et al., 2012). The time-series for this voxel was used for further analysis, resulting in a total of 6 sets of 78 time-series (one for each frequency band, using six classic frequency bands: delta (0.5–4 Hz), theta (4–8 Hz), alpha1 (8–10 Hz), alpha2 (10–13 Hz), beta (13–30 Hz), and gamma (30–48 Hz)). Like in our previous studies we selected five artifact-free epochs of 4096 samples (6.555 s) from these time-series, based on careful visual inspection (PT) to obtain stable results (Douw et al., 2010a, 2010b; Bosboom et al., 2006; Bosma et al., 2008; Douw et al., 2008, 2009; Schoonheim et al., 2013; Stam et al., 2006, 2009; Stoffers et al., 2007; van Dellen et al., 2013). BrainWave software packing was used for this purpose and also for further analyses (version 0.9.58 available from http://home.kpn.nl/stam7883/brainwave.html).

The relative power, averaged over the selected 5 epochs, in every ROI for every subject was calculated using the Fast Fourier Transformation. In addition, the mean power over all ROIs was calculated to yield one mean power value per frequency band in every subject (global relative power). The peak frequency was determined as well. Other properties of the MEG background rhythm were also computed, namely anterior posterior gradients, the amount of diffuse slow wave activity and asymmetry (Lodder and van Putten, 2013). Anterior posterior gradient is the ratio between power in frontal regions and power in all regions for a specific frequency range, often the alpha band. Here we calculated the anterior posterior gradient for both the alpha1 and alpha2 band separately. This gradient is within normal range if it is smaller than 0.4. Diffuse slow wave activity is calculated by the power ratio (Qslow) between Plow (= 2–8 Hz) and Pwide (= 2–25 Hz). Too much or abnormal diffuse slow-wave activity is present when this ratio exceeds 0.6. Asymmetry is obtained by calculating a left-right power ratio for each ROI pair in the frequency range 0.5–12 Hz (Lodder and van Putten, 2013).

### 2.7 Statistical analysis

To compare group differences between MS patients and healthy controls independent *t*-tests and in the absence of normality Mann–Whitney tests were used for all global relative power values, MRI parameters and cognition. Obtained *p*-values for global relative power values were corrected for multiple comparisons with the false discovery rate (i.e. correcting for 6 tests (6 frequency bands)) (Benjamini and Hochberg, 1995). Normality was checked using histogram inspection and Kolmogorov–Smirnov tests of normality. Whenever there were significant differences in global relative power values, power in ROIs were compared between groups by means of permutation analysis as a post-hoc analysis (Nichols and Holmes, 2002). Here a null distribution for between-group differences (independent *t*-test) is derived by permuting group assignment and calculating a t-statistic after each permutation. Other global properties of the MEG rhythm were also statistically analyzed as mentioned above. For analyzing asymmetry differences we used permutation analyses as well.

As another post-hoc analysis, if there were significant differences in global relative power values between MS patients and healthy controls in a specific frequency band, then correlations between mean power and other parameters (EDSS, MRI, and cognition) were computed by means of Spearman's coefficients (2-tailed) for that specific frequency band. These statistical analyses were performed using SPSS for windows v.15. and the permutation analyses were performed in Matlab (R2008b).

## 3 Results

### 3.1 Subject characteristics

Characteristics for the 21 MS patients and 17 healthy controls whose data were used for analysis in the present study are summarized in Table 1. There were no significant differences in age, gender, or educational level between the two groups. Disease duration, EDSS score and MRI lesion load did not differ significantly between male and female patients. Neuropsychological test outcome was not significantly different between MS patients and controls. However, overall cognition Z-scores were significantly lower in male patients compared to female patients (Mann Whitney U: *Z* = − 2.2, *p* = 0.02).

### 3.2 MRI: atrophy measures

NBV and NGMV were significantly lower in the patient group (*t*(36) = − 3.01, *p* = 0.006, *t*(36) = − 2.2, *p* = 0.037, respectively) (Table 1). Mann–Whitney test revealed significantly lower total thalamic volumes (*Z* = − 2.9, *p* = 0.004) in MS patients compared to healthy controls.

### 3.3 Global relative power

**Fig. 1:**
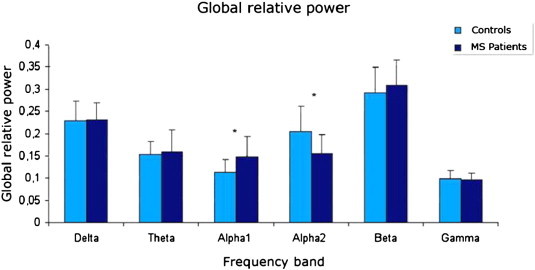
Relative power in the different frequency bands, averaged over all cortical areas (78 AAL ROIs), for patients and healthy controls. Error bars indicate standard deviations. * Indicates significant total power differences between the two groups, which occurred in the alpha1 and alpha2 bands. The patients showed higher alpha1 power and lower alpha2 power. These results were corrected for multiple comparisons with the false discovery rate.

**Fig. 2:**
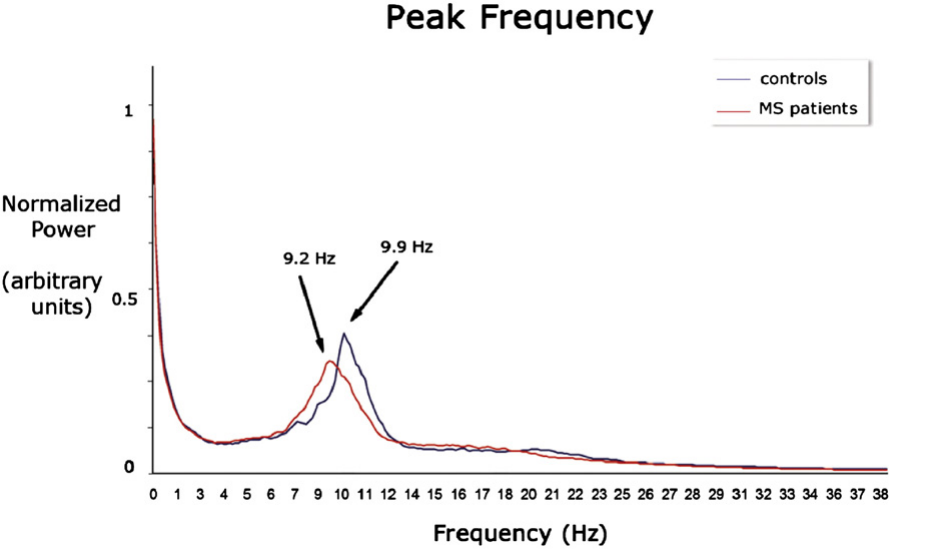
Normalized power spectra, with peak frequency for patients (9.15 Hz) and for controls (9.92 Hz).

Global relative power (averaged over all 78 ROIs) in all the different frequency bands is shown in Fig. 1. Patients showed more mean relative power in the alpha1 band compared to controls (mean patients = 0.15 and mean controls = 0.11, *Z* = − 2.7, *p* = 0.006) and less mean relative power in the alpha 2 band (mean patients = 0.15 and mean controls = 0.21, Z = − 2.7, *p* = 0.007). For the full alpha (8–13 Hz) frequency band global relative power was not different between healthy controls and MS patients (Z = − 1.086 *p* = 0.27). The global relative power in the other bands did not differ significantly between groups. Differences in peak alpha frequency are illustrated in Fig. 2, which shows a significant shift in peak frequency from 9.92 Hz in controls to 9.15 Hz in patients (*t*(36) = 2.138, *p* = 0.039).

### 3.4 Regional relative power

**Fig. 3:**
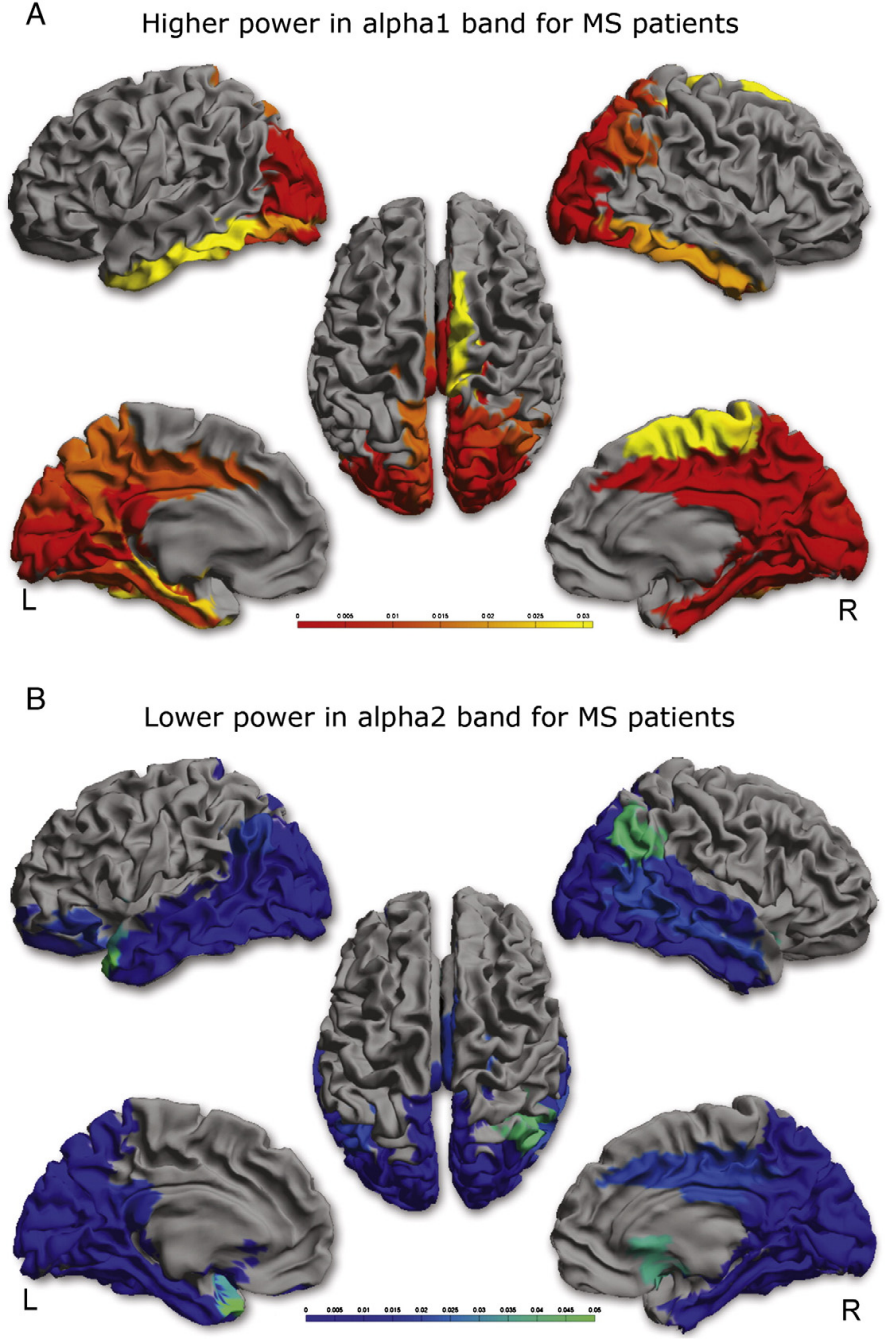
Significant power differences between patients and controls are shown as a color-coded map on a template mesh. Red areas indicate higher power in patients in the alpha1 band (3A), blue areas indicate lower power in patients in the alpha2 band (3B). A scale bar is added to indicate the significance.

Only if global relative power differences were present in a specific frequency band, we compared regional power differences between MS patients and healthy controls as a post hoc analysis. There were significant differences in relative power between patients and controls in the lower and upper alpha band. Fig. 3 displays the results of the post hoc permutation analysis in respectively the alpha1 and alpha2 bands. The nomenclature for the different areas based on automated anatomical labeling (AAL) is given in Appendix A1. Compared to healthy controls, MS patients showed more relative power in the alpha1 band in right and left occipital regions (AAL 22–27, 61–66), inferior temporal regions (AAL 28, 32, 35, 67, 71, 74), medial parietal regions (AAL 21, 56, 59, 60), right frontal regions (AAL 51, 52) and midposterior cingulate regions (37, 38, 76, 77). Additionally, for the alpha2 band, significantly less power was found in MS patients in very similar regions, namely left and right occipital regions (AAL 22–27, 61–66), medial and inferior temporal regions (AAL 28, 31–33, 35, 67, 69–71, 74), parietal regions (AAL 20, 21, 59, 60), midposterior cingulate (AAL 38, 76, 77) and frontal regions (AAL 2, 3, 6, 12, 40, 41).

### 3.5 Other MEG background properties

There was no difference in diffuse slow-wave activity between MS patients and healthy controls nor between asymmetry and anterior–posterior gradients in the alpha1 band. In both MS patients and healthy controls the diffuse slow-wave activity ratio (Q_slow_) was lower than 0.6. In the alpha2 band there was a higher anterior–posterior gradient in MS patients (healthy controls median = 0.25, range = (0.15–0.44), MS patients median = 0.3, range = (0.19–0.42), *Z* = − 1.48 *p* = 0.036).

### 3.6 Correlations between power and cognition, physical disability and MRI

In MS patients, a negative correlation between global relative power in the alpha1 band and overall cognition was found (*r*(19) = − 0.46, *p* = 0.03), driven by information processing speed (*r*(19) = − 0.44, *p* = 0.047). There were no correlations between alpha1 or alpha2 band global relative power and physical disability or MRI parameters in MS patients.

## 4 Discussion

The aim of the present study was to investigate if changes in oscillatory resting-state activity were present in MS and whether these changes were clinically relevant. For this purpose we reconstructed the relative power in several frequency bands for 78 atlas-based regions. Subsequently, relations between relative power and clinical-, cognitive-, and structural-measures were assessed. We found higher mean relative power in the alpha1 band and lower mean relative power in the alpha2 band in MS patients compared to healthy controls. Moreover, cognition and more specifically information processing speed correlated with mean relative power in the alpha1 band.

Here we report for the first time that in early MS there is a shift of the alpha peak towards the slower frequencies. Apart from the power differences at the global level in both lower and upper alpha bands, regional power differences in the same frequency bands were present, mainly localized to occipital, parietal and temporal areas. The observed lower power in the alpha2 band was also strengthened by the finding of a higher anterior–posterior gradient, indicative of a loss of alpha2 power in posterior regions in MS patients. So far, limited research on EEG and MEG power spectra has been performed in MS. Some earlier EEG studies at the sensor-level did find changes in MS spectra compared to controls, namely increased theta power over temporal regions and increased beta power over frontal regions (Colon et al., 1981), increase or hemispheric asymmetry of alpha band power (Facchetti et al., 1994) and increase of beta and gamma band activity over occipital and right frontal areas during an odd-ball task (Vazquez-Marrufo et al., 2008). However the latter study used a small 13-electrode EEG system and subsequently only used the midline electrodes (Fz, Cz, Pz) for analyses. More recent MEG and EEG studies were not able to find resting-state power changes in MS (Cover et al., 2006; Leocani et al., 2000). The use of MEG compared to EEG, in combination with analyses in source-space (which gives an increased signal-to-noise ratio) and a larger sample size, could explain some of the differences between our results and the results from these previous studies.

The power changes in the alpha band seem to be clinically relevant. Power in the alpha1 band correlated negatively with overall cognition and, more specifically, with information processing speed. It has been observed that information processing speed is one of the first affected cognitive domains in MS (Chiaravalloti and DeLuca, 2008). In line with our results, another study on MS revealed that increase of mean alpha frequency was associated with improvement in clinical status (Colon et al., 1981). Therefore it seems relevant to elucidate this relation further in larger MS cohorts and compare power changes in the alpha band between MS patients with preserved information processing speed and impaired information processing speed. Another argument to support further investigation in this relation is the observation that in healthy conditions alpha frequency is related to information processing speed or reaction time (Klimesch, 1999).

We observed a minor but significant slowing of the peak frequency of oscillatory brain activity. The mechanism underlying this slowing remains to be elucidated, particularly since there was no relation between alpha band power changes and structural damage (as determined from MRI measures of NBV, NGMV and T2 lesion load). However, slowing of oscillatory activity has been found for several other neurodegenerative neurological diseases, such as Alzheimer's disease (de Haan et al., 2008; de Waal et al., 2012) and Parkinson's disease (Bosboom et al., 2006; Olde Dubbelink et al., 2012; Stoffers et al., 2007). Therefore slowing of oscillatory activity might be a common pathway in neurodegenerative neurological diseases.

Oscillatory activity at the macroscopic level, such as measured with MEG/EEG, is the result of neuronal interactions at the micro-scale (between individual neurons) and macro-scale (between cortical regions), and involves different types of connections (excitatory and inhibitory). This makes it difficult to elucidate the exact origins of extracranially observed alterations in band-limited oscillatory power (Lopes da Silva, 1991). However, some insight may be gained from computational studies. Two recent studies have used neural mass models in order to explain empirical observations in patients with Alzheimer's disease, and revealed that increased inhibition (Bhattacharya et al., 2011) or disinhibition (de Haan et al., 2012) can cause slowing of oscillatory activity. Therefore a shift in the balance of inhibition/excitation could be of importance in slowing of oscillatory activity in MS as well. Such a shift in the balance of inhibition/excitation can be regarded as a problem of cortical origin.

However white matter changes measured with more advanced and sophisticated MRI measures such as DTI could also be of importance in MS. It has been shown that the integrity (fractional anisotropy values) of white matter tracts such as the corona radiata, posterior thalamic radiation and inferior longitudinal fascicle is positively correlated with the alpha peak frequency (Valdes-Hernandez et al., 2010). In MS we know that the integrity of these tracts is often affected (Harrison et al., 2013; Li et al., 2012). Furthermore, these white matter tracts are predilection sites for white matter lesions (Li et al., 2012). Therefore, the location of white matter lesions (located at white matter tracts going to occipital cortex) could be of more relevance for changes in the alpha peak frequency than overall white matter lesion load itself. Further studies are therefore warranted to elucidate the relation between white matter integrity changes, lesion probability maps and alpha band power changes. Demyelination has also been studied in a computational model of single cell neurons using modified Hodgkin and Huxley models (Coggan et al., 2010). In demyelinated neurons four types of spike behavior could occur after an initial stimulus: delay of a spike, no spike (spike failure), stimulus dependent afterdischarge and spontaneous spiking. This behavior can be explained in terms of the ratio between sodium conductance and the conductance for leakage charge. When leakage out of the neuron is too large due to demyelination this ratio becomes too small which results in spike failure. Increasing this ratio can lead to the other aforementioned behaviors. Failure of spike activity could be responsible for our observed lowering of the frequency of oscillatory activity. Unfortunately, it is still not fully understood how spike observations relate to post synaptic activity observations (main source of LFP/EEG/MEG), therefore future studies are warranted to verify this possible explanation.

The present study has some limitations. Sample size of our study was limited which hampered analysis of medication and gender effects. Limited sample size was partially caused by exclusion of subjects due to inaccurate co-registration and artifacts in the raw data. Future studies will use improved co-registration procedures, as well as a sophisticated artifact removal approach (temporal extension of Signal Space Separation (tSSS)) in order to address these issues (Gross et al., 2013; Taulu and Hari, 2009; Taulu and Simola, 2006; van Dellen et al., 2013). The present study had a cross-sectional design, and we therefore do not know if slowing of oscillatory activity is specific for short disease duration and is still present, or progresses, later on in the disease.

To conclude, slowing of alpha band oscillatory activity occurs in early MS. This seems to be of clinical relevance since these changes were related to information processing speed changes which is one of the first affected cognitive domains in MS. Future larger studies should verify the relation between changes in alpha power and information processing speed, and subsequently with structural changes as well.

## Figures and Tables

**Table:** 

1	Rectus_L	40	Rectus_R
2	Olfactory_L	41	Olfactory_R
3	Frontal_Sup_Orb_L	42	Frontal_Sup_Orb_R
4	Frontal_Med_Orb_L	43	Frontal_Med_Orb_R
5	Frontal_Mid_Orb_L	44	Frontal_Mid_Orb_R
6	Frontal_Inf_Orb_L	45	Frontal_Inf_Orb_R
7	Frontal_Sup_L	46	Frontal_Sup_R
8	Frontal_Mid_L	47	Frontal_Mid_R
9	Frontal_Inf_Oper_L	48	Frontal_Inf_Oper_R
10	Frontal_Inf_Tri_L	49	Frontal_Inf_Tri_R
11	Frontal_Sup_Medial_L	50	Frontal_Sup_Medial_R
12	Supp_Motor_Area_L	51	Supp_Motor_Area_R
13	Paracentral_Lobule_L	52	Paracentral_Lobule_R
14	Precentral_L	53	Precentral_R
15	Rolandic_Oper_L	54	Rolandic_Oper_R
16	Postcentral_L	55	Postcentral_R
17	Parietal_Sup_L	56	Parietal_Sup_R
18	Parietal_Inf_L	57	Parietal_Inf_R
19	SupraMarginal_L	58	SupraMarginal_R
20	Angular_L	59	Angular_R
21	Precuneus_L	60	Precuneus_R
22	Occipital_Sup_L	61	Occipital_Sup_R
23	Occipital_Mid_L	62	Occipital_Mid_R
24	Occipital_Inf_L	63	Occipital_Inf_R
25	Calcarine_L	64	Calcarine_R
26	Cuneus_L	65	Cuneus_R
27	Lingual_L	66	Lingual_R
28	Fusiform_L	67	Fusiform_R
29	Heschl_L	68	Heschl_R
30	Temporal_Sup_L	69	Temporal_Sup_R
31	Temporal_Mid_L	70	Temporal_Mid_R
32	Temporal_Inf_L	71	Temporal_Inf_R
33	Temporal_Pole_Sup_L	72	Temporal_Pole_Sup_R
34	Temporal_Pole_Mid_L	73	Temporal_Pole_Mid_R
35	ParaHippocampal_L	74	ParaHippocampal_R
36	Cingulum_Ant_L	75	Cingulum_Ant_R
37	Cingulum_Mid_L	76	Cingulum_Mid_R
38	Cingulum_Post_L	77	Cingulum_Post_R
39	Insula_L	78	Insula_R

**Table 1:** Descriptive variables for controls and patients.

	Controls (N = 17)	Patients (N = 21)	*p* value
	Mean ± SD	Mean ± SD	
Age	39.8 ± 9.8	41.9 ± 7.7	0.49
Education(1–7)	5.9 ± 1.36	5.4 ± 1.33	0.52
Disease duration		6.8 ± 0.9	–
NGMV (l)	0.84 ± 0.05	0.81 ± 0.04	0.037^a^
NWMV^b^ (l)	0.69 ± 0.03	0.66 ± 0.03	^–^
NBV (l)	1.53 ± 0.07	1.47 ± 0.05	0.006^a^
Total thalamic volume	0.021 ± 0.001	0.019 ± 0.002	0.004^a^
Cognition	0.04 ± 0.64	− 0.19 ± 0.84	0.36
EDSS (1–10)^c^		2 (0–4.5)	–
T1 lesion load (mL)		1.05 ± 0.81	–
T2 lesion load (mL)		2.48 ± 2.03	–

NGMV, normalized gray matter volume; NWMV, normalized white matter volume; NBV, normalized brain volume; EDSS, Expanded Disability Status Scale.

a Indicates significant differences between the two groups.

b NWMV was not used for further analyses; lesion filling was not performed and therefore NWMV was not reliably estimated.

c Indicates median and range.
